# Aminothiazoles inhibit RANKL‐ and LPS‐mediated osteoclastogenesis and PGE_2_ production in RAW 264.7 cells

**DOI:** 10.1111/jcmm.12814

**Published:** 2016-03-14

**Authors:** Anna Kats, Maria Norgård, Zenebech Wondimu, Catalin Koro, Hernán Concha Quezada, Göran Andersson, Tülay Yucel‐Lindberg

**Affiliations:** ^1^ Division of PeriodontologyDepartment of Dental MedicineKarolinska InstitutetHuddingeSweden; ^2^ Division of PathologyDepartment of Laboratory MedicineKarolinska InstitutetHuddingeSweden

**Keywords:** aminothiazole, lipopolysaccharides, prostaglandin E_2_, prostaglandin E synthase, receptor activator of nuclear factor‐κB ligand, RAW 264.7 cells

## Abstract

Periodontitis is characterized by chronic inflammation and osteoclast‐mediated bone loss regulated by the receptor activator of nuclear factor‐κB (RANK), RANK ligand (RANKL) and osteoprotegerin (OPG). The aim of this study was to investigate the effect of aminothiazoles targeting prostaglandin E synthase‐1 (mPGES‐1) on RANKL‐ and lipopolysaccharide (LPS)‐mediated osteoclastogenesis and prostaglandin E_2_ (PGE_2_) production *in vitro* using the osteoclast precursor RAW 264.7 cells. RAW 264.7 cells were treated with RANKL or LPS alone or in combination with the aminothiazoles 4‐([4‐(2‐naphthyl)‐1,3‐thiazol‐2‐yl]amino)phenol (TH‐848) or 4‐(3‐fluoro‐4‐methoxyphenyl)‐*N*‐(4‐phenoxyphenyl)‐1,3‐thiazol‐2‐amine (TH‐644). Aminothiazoles significantly decreased the number of multinucleated tartrate‐resistant acid phosphatase (TRAP)‐positive osteoclast‐like cells in cultures of RANKL‐ and LPS‐stimulated RAW 264.7 cells, as well as reduced the production of PGE_2_ in culture supernatants. LPS‐treatment induced mPGES‐1 mRNA expression at 16 hrs and the subsequent PGE_2_ production at 72 hrs. Conversely, RANKL did not affect PGE_2_ secretion but markedly reduced mPGES‐1 at mRNA level. Furthermore, mRNA expression of TRAP and cathepsin K (CTSK) was reduced by aminothiazoles in RAW 264.7 cells activated by LPS, whereas RANK, OPG or tumour necrosis factor α mRNA expression was not significantly affected. In RANKL‐activated RAW 264.7 cells, TH‐848 and TH‐644 down‐regulated CTSK but not TRAP mRNA expression. Moreover, the inhibitory effect of aminothiazoles on PGE_2_ production was also confirmed in LPS‐stimulated human peripheral blood mononuclear cell cultures. In conclusion, the aminothiazoles reduced both LPS‐ and RANKL‐mediated osteoclastogenesis and PGE_2_ production in RAW 264.7 cells, suggesting these compounds as potential inhibitors for treatment of chronic inflammatory bone resorption, such as periodontitis.

## Introduction

Prostaglandin E_2_ (PGE_2_) plays a central role in chronic inflammatory diseases, including rheumatoid arthritis and periodontitis 1, 2. This pro‐inflammatory mediator is a potent stimulator of tissue destruction and bone resorption, and is reported to be enhanced in gingival crevicular fluid from patients with periodontitis 3, 4, 5. Prostaglandin E_2_ can stimulate the production of matrix metalloproteinases by immune cells and resident cells, including fibroblasts resulting in the degradation of connective tissue and osteoclast‐mediated bone resorption 6, 7.

Prostaglandin E_2_ biosynthesis is regulated by the enzymes phospholipase A_2_, cyclooxygenase (COX) and prostaglandin E synthase (PGES). The terminal PGES enzymes, converting prostaglandin H_2_ to PGE_2_, are present in three different isoforms; glutathione‐dependent membrane‐associated microsomal PGES (mPGES)‐1, glutathione‐independent mPGES‐2 and cytosolic PGES (cPGES) 8. mPGES‐1 is the PGES isoform reported to be induced and co‐regulated with COX‐2, in contrast to the constitutively expressed mPGES‐2 and cPGES 8, 9, 10. In periodontal tissues, our research group have reported that all three isoforms of PGES are expressed in gingival tissues from patients with periodontitis 11. Induction of mPGES‐1 by pro‐inflammatory stimuli, such as lipopolysaccharides (LPS), interleukin‐1β (IL‐1β) or tumour necrosis factor α (TNF‐α), has been described in several cell types 11, 12, 13, 14, 15, 16. In cells involved in bone metabolism, mPGES‐1 expression has shown to be up‐regulated in response to LPS in osteoblasts and mouse bone marrow‐derived macrophages 17, 18. In addition, the enhanced expression of mPGES‐1, COX‐2 as well as PGE_2_ production in response to LPS stimulation has been demonstrated in the myelomonocytic cell line RAW 264.7 12.

Bone remodelling is balanced between bone formation by osteoblasts and bone resorption by osteoclasts under healthy conditions. This system is regulated by the cytokines macrophage colony‐stimulating factor (M‐CSF), receptor activator of nuclear factor‐κB ligand (RANKL), osteoprotegerin (OPG) and RANK. Osteoclastogenesis is mediated by M‐CSF and RANKL released by osteoblasts/osteocytes that activates osteoclast progenitors to mature and fuse into multinucleated osteoclasts 19. In addition, osteoclastogenesis is regulated by inflammatory mediators including cytokines and PGE_2_. Elevated levels of PGE_2_ can shift bone metabolism towards an enhanced osteoclastogenesis by stimulating osteoblasts/osteocytes to increase RANKL production and decrease OPG secretion 5, 20. In addition, PGE_2_ can up‐regulate the expression of RANK, the receptor for RANKL, in osteoclasts 20. It has also been reported that PGE_2_ may exert a dual effect on osteoblasts where low doses of PGE_2_ increases OPG expression and thereby overwhelm RANKL expression, while higher concentrations of PGE_2_ results in the converse effect 21.

Increased levels of PGE_2_ have been observed in gingival crevicular fluid from patients with periodontitis compared to periodontally healthy controls 3, 4. Treatment of periodontal disease with known PGE_2_‐inhibiting drugs, including non‐steroid anti‐inflammatory drugs (NSAID), selective COX‐2 inhibitors, such as celecoxib, or triclosan have shown beneficial effects in terms of reduced gingival inflammation and alveolar bone loss 22, 23, 24. However, long‐term use of selective COX‐2 inhibitors and NSAIDs are associated with unwanted side‐effects as a result of an unspecific inhibition of PGE_2_ production, leading to gastrointestinal and cardiovascular injury 25, 26. Since the enzyme mPGES‐1 acts downstream of COX‐2, it represents an attractive target for new classes of drugs selectively inhibiting PGE_2_ production. Subsequently, intensive research is being performed to identify clinically useful inhibitors for mPGES‐1 27, 28, 29. We reported that aminothiazole derivatives inhibited PGE_2_ in gingival fibroblasts *via* mPGES‐1 without affecting COX‐2, suggesting these compounds as potential novel inhibitors of mPGES‐1 30. Furthermore, we for the first time also demonstrated that local treatment with the aminothiazole TH‐848 reduced alveolar bone loss in a model of experimental periodontitis in rats 30. The aim of this study was to further explore the effect of aminothiazoles on RANKL‐ and LPS‐induced osteoclastogenesis using the osteoclast precursor cell line RAW 264.7.

## Materials and methods

### RAW 264.7 cell cultures

RAW 264.7 cells (American Type Culture Collection, Manassas, VA, USA) were cultured in Minimal Essential Medium Eagle alpha modification (αMEM; Sigma‐Aldrich, St. Louis, MO, USA) supplemented with 10% fetal bovine serum, 2–4 mM L‐glutamine and penicillin (100 U/ml), streptomycin (100 μg/ml) and incubated in 37°C 5% CO_2_ under humidified conditions. The cells were seeded in 6‐, 24‐ or 96‐well plates as previously described 31, and activated by recombinant truncated mouse RANKL at 3 or 30 ng/ml (depending on different batches/activities of the reagent supplied from R&D Systems, Minneapolis, MN, USA) or LPS from *Escherichia coli* at 1 μg/ml (Sigma‐Aldrich). The cells were treated either with different concentrations of the aminothiazoles 4‐([4‐(2‐naphthyl)‐1,3‐thiazol‐2‐yl]amino)phenol (TH‐848) and 4‐(3‐fluoro‐4‐methoxyphenyl)‐*N*‐(4‐phenoxyphenyl)‐1,3‐thiazol‐2‐amine hydrobromide (TH‐644) (Chem‐Bridge Corp., San Diego, CA, USA), exogenous PGE_2_ (Cayman Chemical, Ann Arbor, MI, USA) and Celecoxib (Sigma‐Aldrich) in the absence or presence of RANKL or LPS. The stimulated cells as well as control cells, grown in medium alone, were incubated for 1, 3 or 4 days depending on the analyses described below.

### Human peripheral blood mononuclear cell cultures

Human whole‐blood was collected from healthy volunteers and purchased from the Karolinska University Hospital at Huddinge. The study was approved by the Regional Ethical Review Board in Stockholm. To obtain autologous serum, the blood was centrifuged at 400 × g for 25 min. The plasma was collected and centrifuged at 3000 × g for 15 min. The supernatant was thereafter heat inactivated at 56°C for 30 min. and centrifuged once more for 15 min. Peripheral blood mononuclear cells (PBMCs) were isolated by density gradient separation. Briefly, the plasma‐free blood was diluted 1:1 in PBS without calcium and magnesium and applied on top of Ficoll‐Paque PREMIUM (GE healthcare, Uppsala, Sweden). Following centrifugation at 400 × g for 35 min., the PBMCs were collected at the interface between the density gradient media and PBS. The cells were washed twice with PBS and diluted in base medium containing α‐MEM (Sigma‐Aldrich) supplemented with 15% (v/v) autologous serum, 2–4 mM L‐glutamine and penicillin (100 U/ml), streptomycin (100 μg/ml). Thereafter, the cultures (3–6 × 10^6^ cells/ml) were seeded in 48‐well plates (Sarstedt, Nümbrecht, Gemany), incubated over night at 37°C in 5% CO_2_ and washed with PBS to remove non‐adherent PBMCs. The cell cultures were treated with base medium supplemented with 25 ng/ml M‐CSF in the absence or presence of RANKL (30 ng/ml) or LPS from *E. coli* (0.1 μg/ml) either alone or in combination with TH‐848 (0.2 or 2 μM), TH‐644 (2 or 15 μM) or Celecoxib (1 μM). After 72 hrs, the culture medium was collected and stored in −20°C for PGE_2_ analysis as described below.

### Tartrate‐resistant acid phosphatase staining

RAW 264.7 cells (5–7 × 10^3^ cells/well) were seeded in 24‐well plates and treated with RANKL or LPS in the absence or presence of TH‐848 (0.1, 0.2, 0.3 μM), TH‐644 (10, 15 or 20 μM), exogenous PGE_2_ (0.1, 1 μM) or Celecoxib (1 μM). After 3 days, the culture medium containing the relevant reagents (indicated above) was changed and the cells were allowed to differentiate for additional 1 day and then fixed in 4% paraformaldehyde (Histolab, Gothenburg, Sweden). Tartrate‐resistant acid phosphatase (TRAP) staining was performed with the commercial acid phosphatase leucocyte (TRAP) Kit (Sigma‐Aldrich) according to the manufacturer's instruction. Multinucleated TRAP‐positive cells with ≥3 nuclei were defined as osteoclasts. Osteoclasts were counted under a light microscope by two independent observers. Mean numbers of osteoclasts from three independent experiments were used in the calculations for the half maximal inhibitory concentration (IC_50_), determined by interpolation from the plots of percent inhibition *versus* concentration of the compounds.

### Filamentous actin ring staining

RAW 264.7 cells (6 × 10^3^ cells/well) were seeded in 24‐well plates and treated as described above with RANKL or LPS in the absence or presence of TH‐848 (0.2 μM) or TH‐644 (15 μM). After differentiation, of totally 4–5 days, the cells were fixed in 4% paraformaldehyde (Histolab) and thereafter washed with PBS. For filamentous actin (F‐actin) ring staining, the cells were incubated with fluorescein isothiocyanate‐labelled phalloidin (Sigma‐Aldrich) for 40 min. After washing with PBS, the cell layers were counterstained with 4,6‐diamidino‐2‐phenylindole (DAPI) for 15 min. to visualize the nuclei. F‐actin rings were observed and photographed under a fluorescence microscope using the supported software NIS elements (Nikon Instruments, Amsterdam, The Netherlands).

### Determination of PGE_2_ by enzyme immunoassay

The amount of PGE_2_ in the culture supernatants was detected by a commercial enzyme immunoassay kit (Cayman Chemical) according to the manufacturer's instructions. The results are expressed as PGE_2_ production relative to unstimulated control cells.

### Assessment of mPGES‐1 expression by flow cytometry

RAW 264.7 cells were stimulated with LPS (1 μg/ml) or RANKL (30 ng/ml) in the absence or presence of aminothiazoles, TH‐848 (0.2 μM) or TH‐644 (15 μM) for 16 hrs. Stimulated cells were detached by gentle scraping, washed in PBS containing Ca^2+^ and Mg^2+^, fixed with 1% formaldehyde and permeabilized with 0.1% Saponin in PBS. For intracellular staining, 100 × 10^3^ cells per sample were incubated with primary mPGES‐1 antibodies (polyclonal rabbit; Cayman Chemical) for 45 min. at +4°C, and thereafter detected with fluorescently labelled secondary antibodies (sheep antirabbit conjugated to PE; DakoCytomation, Glostrup, Denmark). For each sample, 10,000 events were acquired and analysed by a FACSVerse flow cytometer using FACSuite software (Becton Dickinson, San Jose, CA, USA). The cells were analysed with regard to mPGES‐1 expression and results are presented as histograms of cell count *versus* fluorescence intensity.

### Quantitative RT‐PCR

RAW 264.7 cells were seeded in 6‐well plate (40 × 10^3^ or 100 × 10^3^ cells/well depending on the experiment) and cultured as described above. After 16 hrs (for mPGES‐1 expression) or 72 hrs (for mPGES‐1 expression, TRAP, cathepsin K (CTSK), RANK, OPG and TNF‐α) of treatment with RANKL or LPS, in the absence or presence of TH‐848 (0.2 μM), TH‐644 (15 μM) or Celecoxib (2 μM), total RNA was isolated from the cells using the commercial RNeasy Mini Kit (Qiagen, Valencia, CA, USA). The amount of total RNA was quantified using a NanoVue Plus Spectrophotometer (GE Healthcare), and first‐strand cDNA was obtained by reverse transcription of 1.0 μg of total RNA using the iScript^™^ cDNA Synthesis Kit (Bio‐Rad, Herkules, CA, USA) in a volume of 20 μl. The quantitative polymerase chain reaction (RT‐qPCR) was performed by amplification of 50 ng of cDNA. Analysis for mPGES‐1, TNF‐α and GAPDH were performed by TaqMan Gene Expression Assays (Applied Biosystems, Foster City, CA, USA) together with Taq‐Man Universal PCR Master Mix (Applied Biosystems) in a volume of 20 μl. RT‐qPCR analysis for TRAP, CTSK, RANK, OPG and GAPDH was performed with specific primers (CyberGene AB, Stockholm, Sweden) and iQ^™^ SYBR^®^ Green Supermix (Bio‐Rad) in a volume of 25 μl. All RT‐qPCR reactions were run in triplicates on the CFX96^™^ Real‐Time PCR detection system (Bio‐Rad), and GAPDH was used as reference gene. Gene expression was calculated according to the ΔΔCt method, where each sample was normalized to the mean of GAPDH. The primer sequences or IDs for TaqMan assays as well as optimal annealing temperatures are presented in Table S1.

### Measurements of CTSK enzyme activity

The effect of aminothiazoles on CTSK enzyme activity was assessed by the commercial CTSK inhibitor screening kit (BioVision Incorporated, Milpitas, CA, USA) according to the manufacturer's instructions. The aminothiazoles TH‐848 and TH‐644 (reconstituted in DMSO) was tested at concentrations of 200 μM towards the human‐purified CTSK enzyme provided in the kit. CTSK inhibitor (FF‐FMK), provided in the kit, was used as positive control. DMSO, added at the same concentrations as the aminothiazoles, was used as negative control. The time‐resolved fluorescence was measured for 60 min. by a FLUOstar Optima microplate reader (BMG Labtech, Ortenberg, Germany) and the slopes for all samples and controls were used to calculate the relative inhibition as described in the kit protocol.

### Cytotoxicity measurements by lactate dehydrogenase release

The cells were seeded in 96‐well plates (1 × 10^3^ cells/well) in triplicates and treated for 72 hrs with TH‐848 (0.1–0.4 μM) or TH‐644 (5–30 μM). Lactate dehydrogenase (LDH) release was measured using the commercial CytoTox 96^®^ Non‐Radioactive Cytotoxicity Assay (Promega, Madison, WI, USA) according to the manufacturer's instructions. Lactate dehydrogenase release in the cell culture supernatants was expressed as percentage of maximum LDH release (*i.e*. cells killed by the lysis solution provided).

### Statistical analysis

All experiments were analysed in duplicates or triplicates, and data representing the mean of at least three independent experiments are presented as stated in the figure legends. Results are expressed as means ± S.D. and Student's *t*‐test (two‐tailed) was used in the statistical analysis. *P* < 0.05 were considered statistically significant.

## Results

### Aminothiazoles decreased osteoclast‐like cell formation

**Figure 1:**
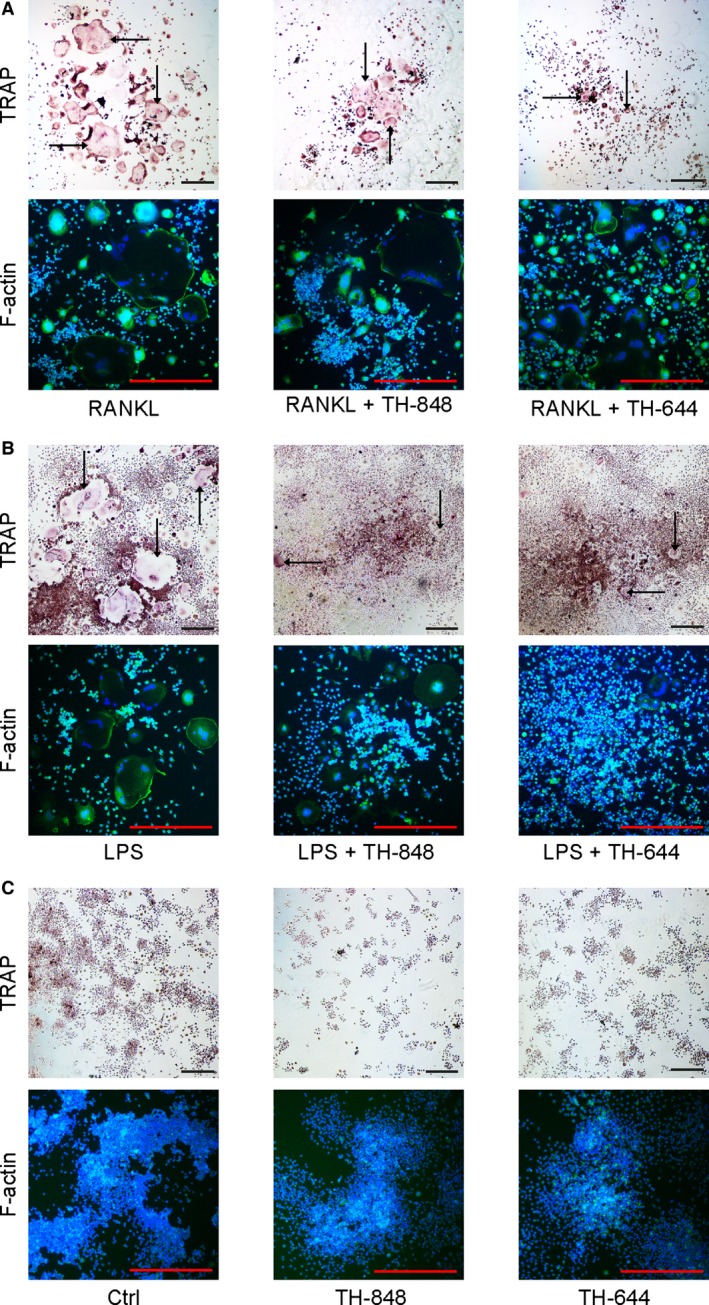
Tartrate‐resistant acid phosphatase (TRAP) and filamentous actin (F‐actin) ring staining. RAW 264.7 cells were treated with receptor activator of nuclear factor‐κB ligand (RANKL; 3 or 30 ng/ml) or lipopolysaccharides (LPS; 1 μg/ml) in the absence or presence of aminothiazoles TH‐848 (0.2 μM) or TH‐644 (15 μM). After 4 days of differentiation, the cell layers were stained for TRAP or F‐actin. (**A**) Cells treated with RANKL alone or in combination with TH‐848 or TH‐644. (**B**) RAW 264.7 cells stimulated with LPS alone or in the presence of TH‐848 or TH‐644. (**C**) Control cells cultured in only medium (Ctrl) or in the presence of TH‐848 or TH‐644. The images are representative of at least three independent experiments, scale bars = 100 μm. Examples of osteoclast‐like cells are marked with arrowheads.

The osteoclast precursor RAW 264.7 cells were stimulated by RANKL or LPS to differentiate into multinucleated osteoclast‐like cells. RAW 264.7 cells were treated for 4 days with RANKL, LPS or medium (Ctrl) alone or in combination with the aminothiazoles TH‐848 or TH‐644, and thereafter stained for TRAP or F‐actin (as shown in Fig. 1A–C). Cells positive for TRAP and containing ≥3 nuclei were defined as osteoclast‐like cells and counted by two independent observers. Treatment with the aminothiazoles TH‐848 ≥0.2 μM and TH‐644 ≥15 μM significantly (*P* < 0.05) inhibited both RANKL‐ and LPS‐stimulated RAW 264.7 cell differentiation to TRAP‐positive osteoclast‐like cells (Table 1). Interestingly both the total number of osteoclast‐like cells as well their individual sizes appeared to be reduced by the aminothiazoles, although a more pronounced inhibitory effect was observed in cells treated with LPS as compared to RANKL (Fig. 1, Table 1). The IC_50_ values for TH‐848 and TH‐644 were 0.20 ± 0.08 μM and 12.9 ± 3.2 μM, respectively, based on three independent experiments stimulated by RANKL. When the cells were treated with higher concentrations of TH‐848 (≥0.4 μM) or TH‐644 (≥30 μM) osteoclastogenesis was completely abolished and no multinucleated cells could be observed (data not shown). Similar to the aminothiazoles, the selective COX‐2 inhibitor, Celecoxib (1 μM) known to inhibit PGE_2_, also significantly (*P* < 0.05) decreased the number of TRAP‐positive osteoclasts in cultures treated with RANKL (Table 1). To further confirm the role of PGE_2_ in osteoclastogenesis, we also investigated the effect of exogenous PGE_2_ (0.1 and 1 μM) on RANKL‐stimulated differentiation of RAW 264.7 cells into TRAP‐positive osteoclast‐like cells. Addition of PGE_2_ in the presence of RANKL increased the formation of TRAP‐positive multinucleated cells by approximately 50% at 0.1 μM (Table 1).

**Table 1:** Numbers of TRAP‐positive osteoclast‐like cells in cultures of RAW 264.7 cells differentiated with RANKL (3 ng/ml) or LPS (1 μg/ml) in the absence or presence of TH‐848 (0.1–0.3 μM), TH‐644 (10–20 μM), Celecoxib (1 μM) or exogenous PGE_2_ (0.1 and 1 μM) for 4 days

Treatment	Concentration (μM)	Osteoclast‐like cell number ± S.D. (relative to RANKL)	Osteoclast‐like cell number ± S.D. (relative to LPS)
RANKL		1.00 ± 0.10	
LPS			1.00 ± 0.13
TH‐848	0.1	0.86 ± 0.43	
	0.2	0.46 ± 0.22^a^	0.19 ± 0.07^b^
	0.3	0.34 ± 0.28^a^	
TH‐644	10	0.80 ± 0.34	
	15	0.40 ± 0.24^a^	0.22 ± 0.12^b^
	20	0.13 ± 0.11^a^	
Celecoxib	1	0.64 ± 0.04^a^	
PGE_2_	0.1	1.42 ± 0.15^a^	
	1	1.49 ± 0.08^a^	

a *P* < 0.05 compared to RANKL.

b *P* < 0.05 compared to LPS.

TRAP‐positive cells containing ≥3 nuclei were counted as osteoclast‐like cells by two observers. Data shown represent ≥3 independent experiments performed in duplicates or triplicates relative to the mean of only RANKL or LPS stimulation.

TRAP: tartrate‐resistant acid phosphatase; RANKL: receptor activator of nuclear factor‐κB ligand; LPS: lipopolysaccharides; PGE_2_: prostaglandin E_2_.

Cytotoxicity, assessed by the release of LDH into the medium, did not reveal any increase in LDH from cells treated with TH‐848 (0.1–0.4 μM) or TH‐644 (5–30 μM). LDH release, relative to maximum release (completely lysed cells), was 21.9 ± 1.4% for control cells incubated with culture medium alone, 11.7 ± 2.9% for cells treated with TH‐848 (0.4 μM) and 20.1 ± 4.7% for cells treated with TH‐644 (30 μM) (Fig. not shown).

### PGE_2_ production is reduced by the aminothiazoles

**Figure 2:**
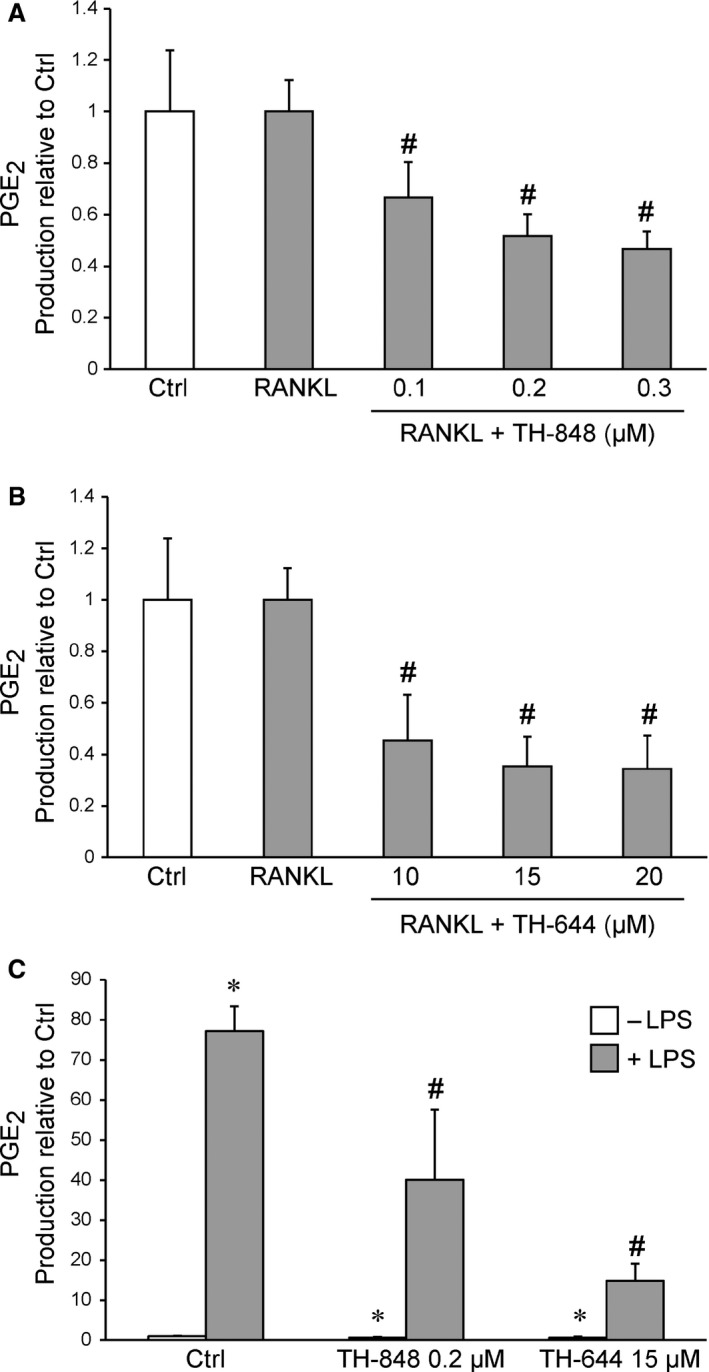
Production of prostaglandin E_2_ (PGE_2_) in cultures of receptor activator of nuclear factor‐κB ligand (RANKL)‐ or lipopolysaccharides (LPS)‐induced RAW 264.7 cells treated with aminothiazoles. RAW 264.7 cells were treated with RANKL (3 ng/ml) or LPS (1 μg/ml) alone or in combination with the aminothiazoles for 72 hrs. (**A** and **B**) PGE_2_ production in the culture supernatants of untreated cells (Ctrl), treated with RANKL alone or in the presence of (**A**) TH‐848 (0.1, 0.2, 0.3 μM) or (**B**) TH‐644 (10, 15, 20 μM). (**C**) RAW 264.7 cells treated with or without LPS (Ctrl) in the presence or absence of TH‐848 (0.2 μM) or TH‐644 (15 μM). The results are expressed as PGE_2_ production relative to Ctrl and represent at least three independent experiments analysed in triplicates. *P < 0.05 compared to Ctrl cells treated with medium alone, ^#^ *P* < 0.05 compared to RANKL‐ or LPS‐treated cells.

The production of PGE_2_ was measured in the culture supernatants of RAW 264.7 cells treated with medium, RANKL or LPS alone or in combination with TH‐848 or TH‐644. The production of PGE_2_ was not affected by RANKL (3 ng/ml) treatment (Fig. 2A and B). However, when the cells were treated with RANKL in combination with the aminothiazoles TH‐848 (0.1, 0.2, 0.3 μM) or TH‐644 (10, 15, 20 μM), the PGE_2_ production was decreased significantly (*P* < 0.05) compared to RANKL‐treated cells (Fig. 2A and B). The IC_50_ values for inhibition of PGE_2_ by TH‐848 and TH‐644 in RANKL‐treated cells were 0.24 ± 0.05 μM and 10.4 ± 1.9 μM respectively. In addition, the effect of aminothiazoles was also investigated on LPS‐stimulated cells. In contrast to RANKL, LPS significantly (*P* < 0.05) increased PGE_2_ production compared to control cells. The aminothiazoles TH‐848 (0.2 μM) and TH‐644 (15 μM) significantly (*P* < 0.05) prevented the LPS‐stimulated PGE_2_ production (Fig. 2C). The effect on PGE_2_ by the aminothiazoles corresponded with the decreased number of multinucleated osteoclast‐like cells positively stained for TRAP (Table 1).

**Figure 3:**
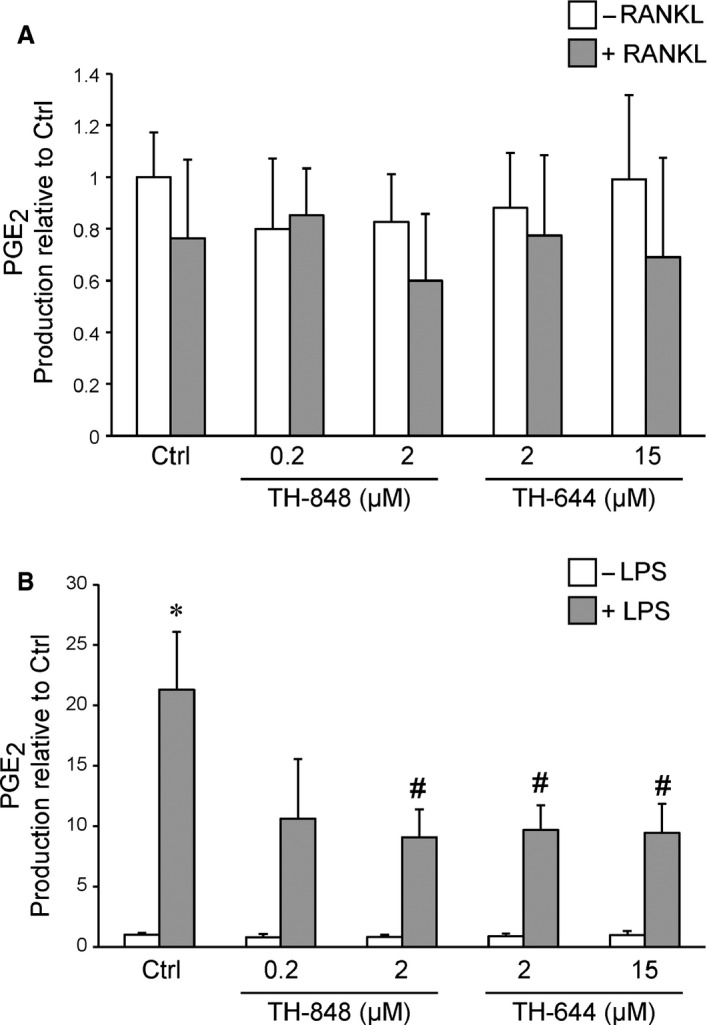
Effect of aminothiazoles on prostaglandin E_2_ (PGE_2_) production in cultures of receptor activator of nuclear factor‐κB ligand (RANKL) or lipopolysaccharides (LPS)‐stimulated human peripheral blood mononuclear cells (PBMC). PBMCs were treated with RANKL (30 ng/ml) or LPS (0.1 μg/ml) alone or in combination with the aminothiazoles for 72 hrs. (**A**) PGE_2_ production in the culture supernatants of PBMCs treated with or without RANKL (Ctrl) in the presence or absence of TH‐848 (0.2 or 2 μM) or TH‐644 (2 or 15 μM). (**B**) PBMCs treated with or without LPS (Ctrl) in the presence or absence of TH‐848 (0.2 or 2 μM) or TH‐644 (2 or 15 μM). The results are expressed as PGE_2_ production relative to untreated Ctrl cells and represent at least three independent experiments analysed in triplicates. *P < 0.05 compared to Ctrl cells treated with medium alone, ^#^ *P* < 0.05 compared to RANKL‐ or LPS‐treated cells.

Prostaglandin E_2_ levels were also measured in cell cultures of human PBMCs stimulated with RANKL or LPS alone or in combination with TH‐848, TH‐644 or Celecoxib for 72 hrs. The results showed that neither RANKL nor the aminothiazoles alone affected the levels of PGE_2_ in PBMCs (Fig. 3A). LPS treatment, on the other hand, significantly (*P* < 0.05) increased the PGE_2_ production as compared to control cells treated with only medium. The aminothiazoles TH‐848 (2 μM) and TH‐644 (2 and 15 μM) significantly (*P* < 0.05) reduced the LPS‐stimulated PGE_2_ production (Fig. 3B). Similar to aminothiazoles, the selective COX‐2 inhibitor, Celecoxib (1 μM), in combination with LPS significantly (*P* < 0.05) decreased PGE_2_ production compared to cells stimulated with LPS only (8.33 ± 0.24 and 21.3 ± 4.77 respectively). However, Celecoxib in combination with RANKL did not affect the PGE_2_ levels as compared to cells treated with RANKL alone (0.73 ± 0.21 and 0.76 ± 0.30 respectively).

### Effect of aminothiazoles on mPGES‐1 protein and mRNA expression

**Figure 4:**
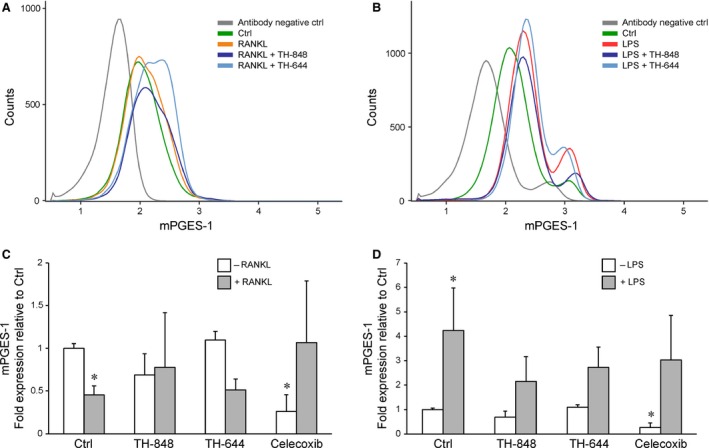
Prostaglandin E synthase‐1 (mPGES‐1) protein and mRNA expression in response to TH‐848 and TH‐644 treatment. RAW 264.7 cells were stimulated with (**A** and **C**) receptor activator of nuclear factor‐κB ligand (RANKL; 30 ng/ml) or (**B** and **D**) lipopolysaccharides (LPS; 1 μg/ml) alone or in combination with aminothiazoles TH‐848 (0.2 μM), TH‐644 (15 μM) or (**C** and **D**) Celecoxib (2 μM) for 16 hrs. (**A** and **B**) Protein expression of mPGES‐1, analysed by flow cytometry, presented as histograms of cell counts *versus* logarithmic fluorescence intensity. Results are representative of ≥3 independent experiments. (**C** and **D**) mPGES‐1 mRNA expression, analysed by RT‐qPCR, expressed as fold expression normalized to glyceraldehyde‐3‐phosphate dehydrogenase (GAPDH) relative to unstimulated cells (Ctrl). Results shown represent at least three independent experiments analysed in triplicates. *P < 0.05 compared to Ctrl.

The effect of aminothiazoles (TH‐848 and TH‐644) was studied on intracellular mPGES‐1 protein expression in RANKL‐ or LPS‐stimulated RAW 264.7 cells cultured for 16 hrs. Treatment of the cells with RANKL, in the absence or presence of aminothiazoles, did not significantly affect mPGES‐1 protein expression as compared to non‐stimulated corresponding control cells (Fig. 4A). In contrast to RANKL, LPS stimulation increased mPGES‐1 protein expression, although neither TH‐848 nor TH‐644 affected the LPS‐induced mPGES‐1 expression (Fig. 4B).

Microsomal PGES‐1 mRNA expression was significantly down‐regulated by RANKL and up‐regulated by LPS at 16 hrs (Fig. 4C and D). The aminothiazoles did not significantly affect the mPGES‐1 expression neither in RANKL‐ nor in LPS‐treated cultures (Fig. 4C and D), although a trend towards reduced mPGES‐1 expression was observed in LPS‐stimulated cells (Fig. 4C). Similarly as with the aminothiazoles, the COX‐2 inhibitor Celecoxib did not affect the mPGES‐1 expression in RANKL‐ or LPS‐treated cells, although Celecoxib treatment significantly down‐regulated the expression of mPGES‐1 compared to control cells treated with medium alone (Fig. 4C and D).

The effect of aminothiazoles was also investigated after 72 hrs on mPGES‐1 mRNA expression, at an intermediate stage of osteoclast formation. The stimulatory effect of LPS at 16 hrs was not observed after 72 hrs of treatment, and furthermore no effect of the aminothiazoles on mPGES‐1 mRNA expression was observed neither in LPS‐treated or control cells (Table 2). In response to RANKL treatment, mPGES‐1 expression continued to decrease also after 72 hrs of incubation, but no inhibitory effect by aminothiazoles was observed on mPGES‐1 mRNA expression (Table 3).

**Table 2:** The effect of aminothiazoles on LPS‐stimulated mPGES‐1, TRAP, CTSK, RANK, OPG and TNF‐α mRNA expression

Treatment	Gene (mean expression ± S.D. relative to Ctrl)					
	mPGES‐1	TRAP	CTSK	RANK	OPG	TNF‐α
Ctrl	1.00 ± 0.06	1.00 ± 0.09	1.00 ± 0.21	1.00 ± 0.13	1.00 ± 0.18	1.00 ± 0.22
LPS	0.98 ± 0.10	49.1 ± 19.6^a^	235 ± 88.4^a^	0.71 ± 0.22	0.13 ± 0.06^a^	3.94 ± 0.67^a^
LPS + TH‐848	1.17 ± 0.24	9.07 ± 3.57^a^^,^ b	18.5 ± 10.6^a^^,^ b	0.69 ± 0.24	0.37 ± 0.28^a^	3.88 ± 0.30^a^
LPS + TH‐644	1.10 ± 0.34	16.9 ± 5.60^a^^,^ b	54.9 ± 7.71^a^^,^ b	0.40 ± 0.16^a^	0.20 ± 0.12^a^	4.91 ± 1.72^a^

a *P* < 0.05 relative to Ctrl.

b *P* < 0.05 relative to LPS.

RAW 264.7 cells were stimulated by LPS (1 μg/ml) alone or in the presence of TH‐848 (0.2 μM) or TH‐644 (15 μM) for 72 hrs. Control cells (Ctrl) were treated with medium alone. mRNA expression of mPGES‐1, TRAP, CTSK, RANK and OPG and TNF‐α was analysed by RT‐qPCR and normalized to GAPDH. Results are presented as mean fold change ± S.D. relative to Ctrl. Data shown represent at least three independent experiments analysed in triplicates.

LPS: lipopolysaccharides; mPGES‐1: prostaglandin E synthase‐1; TRAP: tartrate‐resistant acid phosphatase; CTSK: cathepsin K; RANK: receptor activator of nuclear factor‐κB; OPG: osteoprotegerin; TNF‐α: tumour necrosis factor α; GAPDH: glyceraldehyde‐3‐phosphate dehydrogenase.

**Table 3:** The effect of aminothiazoles on RANKL‐stimulated mPGES‐1, TRAP, CTSK, RANK, OPG and TNF‐α mRNA expression

Treatment	Gene (mean expression ± S.D. relative to Ctrl)					
	mPGES‐1	TRAP	CTSK	RANK	OPG	TNF‐α
Ctrl	1.00 ± 0.06	1.00 ± 0.34	1.00 ± 0.16	1.00 ± 0.11	1.00 ± 0.29	1.00 ± 0.23
RANKL	0.10 ± 0.02^a^	909 ± 440^a^	3030 ± 1077^a^	1.27 ± 0.15	0.76 ± 0.48	0.55 ± 0.27
RANKL + TH‐848	0.14 ± 0.04^a^	840 ± 472^a^	759 ± 460^a^^,^ b	1.35 ± 0.44	1.23 ± 0.60	0.33 ± 0.15^a^
RANKL + TH‐644	0.14 ± 0.004^a^	820 ± 484^a^	823 ± 187^a^^,^ b	1.13 ± 0.24	1.01 ± 0.71	0.62 ± 0.44

a *P* < 0.05 relative to Ctrl.

b *P* < 0.05 relative to RANKL.

RAW 264.7 cells were treated with medium (Ctrl) or RANKL (3 ng/ml) alone or in the presence of TH‐848 (0.2 μM) or TH‐644 (15 μM) for 72 hrs. mRNA expression of mPGES‐1, TRAP, CTSK, RANK and OPG and TNF‐α was analysed by RT‐qPCR and normalized to GAPDH. Results are presented as mean fold change ± S.D. relative to Ctrl. Data shown represent three independent experiments analysed in triplicates.

RANKL: receptor activator of nuclear factor‐κB ligand; mPGES‐1: prostaglandin E synthase‐1; TRAP: tartrate‐resistant acid phosphatase; CTSK: cathepsin K; RANK: receptor activator of nuclear factor‐κB; OPG: osteoprotegerin; TNF‐α: tumour necrosis factor α; GAPDH: glyceraldehyde‐3‐phosphate dehydrogenase.

### Effect of aminothiazoles on TRAP, CTSK, RANK, OPG and TNF‐α mRNA expression

Expression of the osteoclast markers TRAP and CTSK, receptor RANK, decoy receptor OPG as well as the pro‐inflammatory cytokine TNF‐α was investigated at the mRNA level in RAW 264.7 cells treated with LPS or RANKL.

In cultures stimulated with LPS, the expression of TRAP and CTSK was strongly up‐regulated (Table 2). The aminothiazoles TH‐848 and TH‐644 decreased significantly the expression of both TRAP and CTSK in LPS‐stimulated cultures (Table 2). Expression of RANK showed a non‐significant tendency to be reduced by LPS alone or in combination with aminothiazoles (Table 2). On the other hand LPS down‐regulated the OPG mRNA expression, which was not further affected by aminothiazoles (Table 2). Furthermore, LPS up‐regulated TNF‐α mRNA expression, which was not affected by aminothiazoles (Table 2).

In cultures stimulated with RANKL, the mRNA expression of TRAP and CTSK was strongly up‐regulated (Table 3). Treatment with aminothiazoles significantly decreased CTSK, but not TRAP, mRNA expression (Table 3). The expression of RANK, OPG and TNF‐α was not significantly affected by RANKL alone or in combination with the aminothiazoles (Table 3).

### Effect of aminothiazoles on CTSK activity

**Figure 5:**
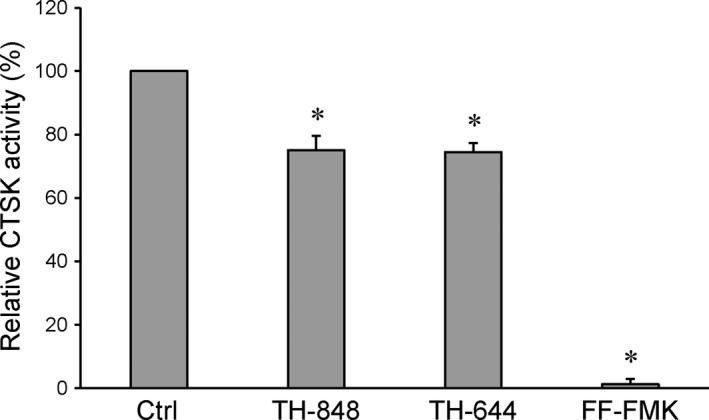
Effect of aminothiazoles on cathepsin K (CTSK) activity. CTSK activity was measured in the presence or absence of TH‐848 (200 μM) or TH‐644 (200 μM). The CTSK inhibitor FF‐FMK was used as positive control. Dimethyl sulfoxide (DMSO) at the same concentrations as the aminothiazoles was added to the enzyme control (Ctrl). The results were determined as CTSK activity (%) relative to Ctrl and represent three independent experiments analysed in triplicates. *P < 0.05 compared to Ctrl.

To further evaluate the effect of aminothiazoles on CTSK, *in vitro* enzyme activity assay was performed in the next series of experiments. The results showed that TH‐848 and TH‐644 decreased the activity by approximately 25% as demonstrated in Figure 5. The specific inhibitor of CTSK, FF‐FMK, used as positive control, completely abolished the activity of CTSK by 98.8 ± 1.6% (Fig. 5).

## Discussion

We have recently reported that aminothiazoles inhibit mPGES‐1 and PGE_2_ *in vitro* as well as reduce alveolar bone loss in ligature‐induced experimental periodontitis *in vivo* 30. In the current study, we used the well‐documented model of osteoclastogenesis, the macrophage cell line RAW 264.7 32, to further investigate the effect of aminothiazoles on osteoclast differentiation. The novel finding of this study is that the aminothiazoles inhibited osteoclastogenesis and decreased PGE_2_ production in RANKL‐ and LPS‐stimulated RAW 264.7 murine macrophage cells.

The number of multinucleated osteoclast‐like cells, positively stained for TRAP, was decreased by aminothiazoles in RANKL‐ as well as in LPS‐stimulated RAW 264.7 cells, suggesting inhibition of osteoclastogenesis by these compounds. The inhibitory effect of aminothiazoles, assessed by counts of TRAP‐positive osteoclast‐like cells and F‐actin staining, was more pronounced in LPS‐stimulated cells compared to RANKL‐treated cells. This suggests that the inhibitory effects of aminothiazoles may act through different mechanisms induced by LPS and RANKL mediated osteoclastogenesis. In line with these results, osteoclast‐like cells formed by RANKL or LPS express different phenotypes suggesting different regulation of osteoclast differentiation during physiological and inflammatory conditions 33. To further explore the inhibitory effect of aminothiazoles on osteoclastogenesis in relation to PGE_2_ production, we determined the production of PGE_2_ in culture supernatants of RAW 264.7 cells. RANKL had no effect on PGE_2_ content alone. However, the aminothiazoles dose‐dependently reduced PGE_2_ production in the RANKL‐treated cultures. In contrast to RANKL, LPS strongly increased the PGE_2_ production, which was reduced by the aminothiazoles TH‐848 and TH‐644. In agreement with RAW 264.7 cells, the stimulatory effect of LPS on PGE_2_ production was decreased by aminothiazoles also in PBMC cultures. However, PGE_2_ levels were not affected neither by aminothiazoles alone or in combination with RANKL. The inhibitory effect of aminothiazoles on LPS‐stimulated PGE_2_ production, both in RAW 264.7 and PBMC cells, may partly explain the more pronounced reduction in osteoclast‐like cells in response to LPS, highlighting the role of PGE_2_ in osteoclastogenesis. These findings are also consistent with our previous results demonstrating decreased PGE_2_ production by aminothiazoles in IL‐1β‐stimulated human gingival fibroblasts and rat gingival fibroblasts 30. Moreover, the very similar dose‐dependency (IC_50_) for inhibition by the aminothiazoles TH‐848 and TH‐644 of RANKL‐dependent osteoclastogenesis in comparison to PGE_2_ production is not inconsistent with a causal relationship between these parameters. In the next series of experiments, the effect of aminothiazoles on mRNA and protein expression of mPGES‐1 was investigated. Aminothiazoles did not significantly affect mPGES‐1 expression neither in LPS‐ nor in RANKL‐treated RAW 264.7 cells. These results are in agreement with our previous study reporting an inhibitory effect by the aminothiazoles on mPGES‐1 enzyme activity rather than protein or mRNA expression 30. However, the mechanisms how LPS induce whereas RANKL reduce mPGES‐1 mRNA expression remains to be clarified.

To further explore the inhibitory effect of aminothiazoles on osteoclastogenesis, mRNA expression of genes involved in bone metabolism was analysed. The enzymes TRAP and CTSK, predominantly expressed by osteoclasts have central roles in the modification and degradation of bone matrix 34, 35. In the current study, both TRAP and CTSK was down‐regulated by the aminothiazoles in LPS‐stimulated cultures of RAW 264.7 cells. Curiously, TRAP mRNA expression was not affected significantly in RANKL‐stimulated cultures. In agreement with our results, the known inhibitor of mPGES‐1, curcumin 36, 37, is reported to decrease both TRAP and CTSK expression *in vivo* 38. Similar to LPS, the expression of CTSK was also down‐regulated by the aminothiazoles in RANKL‐stimulated cultures leading us to further investigate the effect of aminothiazoles on the enzyme activity of CTSK. Our results that the aminothiazoles slightly inhibited CTSK activity may partly explain the inhibitory effect of aminothiazoles on alveolar bone loss observed in experimental periodontitis 30. This suggestion is further supported by Hao *et al*. reporting that inhibition of CTSK may reduce both osteoclast function and inflammation in a mouse model of experimental periodontitis 39.

An interesting observation was that LPS, in contrast to RANKL, showed a significant inhibitory effect on OPG mRNA levels. It can be speculated that the reduction in the osteoclastogenesis inhibitor OPG, possibly in combination with increased expression of TNF‐α and PGE_2_, act synergistically to induce osteoclast formation in the presence of LPS.

Inhibitors of PGE_2_ currently used for clinical treatment of inflammation and bone resorption, including NSAIDs and selective COX‐2 inhibitors, act mainly through inhibition of the COX enzymes acting upstream of mPGES‐1 40. In the present study, we also observed that the selective COX‐2 inhibitor Celecoxib, similar to the aminothiazoles, reduced osteoclast differentiation in the cultures as previously reported 41. In addition, it has been reported that PGE_2_ production of RANKL‐stimulated RAW 264.7 cells was decreased by Celecoxib 41, suggesting that the inhibitory effect of Celecoxib on osteoclastogenesis involves PGE_2_. These results are in agreement with our findings that exogenous PGE_2_ increased the number of osteoclasts in cultures of RANKL‐stimulated RAW 264.7 cells, which has also been demonstrated in previous studies 41, 42. Both NSAIDs and selective COX‐2 inhibitors are known to cause side‐effects, such as gastrointestinal injury and increased risk for cardiovascular events, because of unspecific inhibition of PGE_2_ synthesis 25, 43. Since the discovery of the side‐effects associated with COX inhibitors, research has focused on the terminal enzyme mPGES‐1 as an attractive therapeutic target for more specific inhibition of PGE_2_ 29, 44. Several mPGES‐1 inhibitors have been identified and tested *in vitro* and in experimental models, and a few of these compounds have been reported to inhibit mPGES‐1 in osteoblasts and osteoclast precursors 45, 46, 47. Thus, to our knowledge, there are no selective mPGES‐1 inhibitors available for clinical treatment of inflammatory diseases 48.

Our study showed that the anti‐inflammatory compounds aminothiazoles inhibit the osteoclastogenesis in RAW 264.7 cells and reduce the production of PGE_2_ in RAW 264.7 as well as in human PBMC cultures. Current results together with our previous novel findings that aminothiazoles inhibit PGE_2_ in human gingival fibroblasts as well as decrease alveolar bone resorption in experimental periodontitis in rats 30, suggests that these compounds have the potential to be used for the treatment of bone resorption in chronic inflammatory diseases, such as periodontitis.

## Conflicts of interest

The authors confirm that there are no conflicts of interest.

## Author contribution

AK, MN, GA, TYL designed the research study; AK, MN, ZW, CK performed the experiments; AK, MN, ZW, HCQ, GA, TYL analysed and interpreted the data; AK and TYL wrote the paper; GA critically revised the paper. All authors approved the final version of the paper.

## Supporting information


**Table S1** Primers used in the mRNA expression analysis of glyceraldehyde‐3‐phosphate dehydrogenase (GAPDH), cathepsin K (CTSK), tartrate‐resistant acid phosphatase (TRAP), receptor activator of nuclear factor‐κB (RANK), osteoprotegerin (OPG), prostaglandin E synthase‐1 (mPGES‐1), tumour necrosis factor α (TNF‐α).
