# Supplementary material for: Aminothiazoles inhibit RANKL‐ and LPS‐mediated osteoclastogenesis and PGE 2 production in RAW 264.7 cells
Source: J Cell Mol Med. 2016 Mar 14;20(6):1128–38. doi: 10.1111/jcmm.12814 (PMC4882984; doi:10.1111/jcmm.12814)
Supplement: Supplementary file 1 — Table S1 Primers used in the mRNA expression analysis of glyceraldehyde‐3‐phosphate dehydrogenase (GAPDH), cathepsin K (CTSK), tartrate‐resistant acid phosphatase (TRAP), receptor activator of nuclear factor‐κB (RANK), osteoprotegerin (OPG), prostaglandin E synthase‐1 (mPGES‐1), tumour necrosis factor α (TNF‐α). [file JCMM-20-1128-s001.docx]

| Gene Name | Annealing temperature | Assay ID | Sense 5′-3′ | Antisense 5′-3′ |
| --- | --- | --- | --- | --- |
| GAPDH | 62 °C |  | AAGGCTGTGGGCAAGGTCAT | TGATGTCATCATACTTGGCAGGTT |
| CTSK | 60 °C |  | GTTGTATGTATAACGCCACGGC | CTTTCTCGTTCCCCACAGGA |
| TRAP | 62 °C |  | CAGCCCAAAATGCCTCGA | GCTTTTTGAGCCAGGACAGC |
| RANK | 57 °C |  | TGGCTACCACTGGAACTCAGAC | TGCACACCGTATCCTTGTTGAG |
| OPG | 59 °C |  | CATCCAAGACATTGACCTCTGTGA | TCTCTTCTGGGCTGATCTTCTTCC |
| GAPDH | 60 °C | Mm99999915_g1 |  |  |
| mPGES-1 | 60 °C | Mm00452105_m1 |  |  |
| TNFα | 60 °C | Mm00443260_g1 |  |  |

**Supplementary Table 1** Primers used in the mRNA expression analysis of glyceraldehyde-3-phosphate dehydrogenase (GAPDH), cathepsin K (CTSK), tartrate resistant acid phosphatase (TRAP), receptor activator of nuclear factor-κB (RANK), osteoprotegerin (OPG), prostaglandin E synthase-1 (mPGES-1), tumour necrosis factor α (TNFα).
